# The costs and financing needs of delivering Kenya’s primary health care service package

**DOI:** 10.3389/fpubh.2023.1226163

**Published:** 2023-10-12

**Authors:** Agatha Olago, Christian Suharlim, Salim Hussein, David Njuguna, Stephen Macharia, Rodrigo Muñoz, Marjorie Opuni, Hector Castro, Clarisse Uzamukunda, Damian Walker, Sarah Birse, Elizabeth Wangia, Colin Gilmartin

**Affiliations:** ^1^Kenya Ministry of Health, Department of Primary Health Care, Nairobi, Kenya; ^2^Management Sciences for Health, Medford, MA, United States; ^3^Management Sciences for Health, Health Economics and Financing, Arlington, VA, United States; ^4^Kenya Ministry of Health, Health Economist, Nairobi, Kenya; ^5^Kenya Ministry of Health, Director of Planning, Chief Economist and Head of Planning, Nairobi, Kenya; ^6^Sistemas Integrales, Santiago, Chile; ^7^Independent Consultant, Lausanne, Switzerland; ^8^Independent Consultant, Kigali, Rwanda; ^9^Kenya Ministry of Health, Department of Health Financing, Nairobi, Kenya

**Keywords:** actual cost, normative cost, primary health care, universal health coverage, Kenya

## Abstract

**Introduction:**

For many Kenyans, high-quality primary health care (PHC) services remain unavailable, inaccessible, or unaffordable. To address these challenges, the Government of Kenya has committed to strengthening the country’s PHC system by introducing a comprehensive package of PHC services and promoting the efficient use of existing resources through its primary care network approach. Our study estimated the costs of delivering PHC services in public sector facilities in seven sub-counties, comparing actual costs to normative costs of delivering Kenya’s PHC package and determining the corresponding financial resource gap to achieving universal coverage.

**Methods:**

We collected primary data from a sample of 71 facilities, including dispensaries, health centers, and sub-county hospitals. Data on facility-level recurrent costs were collected retrospectively for 1 year (2018–2019) to estimate economic costs from the public sector perspective. Total actual costs from the sampled facilities were extrapolated using service utilization data from the Kenya Health Information System for the universe of facilities to obtain sub-county and national PHC cost estimates. Normative costs were estimated based on standard treatment protocols and the populations in need of PHC in each sub-county.

**Results and discussion:**

The average actual PHC cost per capita ranged from US$ 9.3 in Ganze sub-county to US$ 47.2 in Mukurweini while the normative cost per capita ranged from US$ 31.8 in Ganze to US$ 42.4 in Kibwezi West. With the exception of Mukurweini (where there was no financial resource gap), closing the resource gap would require significant increases in PHC expenditures and/or improvements to increase the efficiency of PHC service delivery such as improved staff distribution, increased demand for services and patient loads per clinical staff, and reduced bypass to higher level facilities. This study offers valuable evidence on sub-national cost variations and resource requirements to guide the implementation of the government’s PHC reforms and resource mobilization efforts.

## 1. Introduction

The Government of Kenya (GoK) has committed to achieving universal health coverage (UHC) to improve access to high quality and affordable health care for its population. In addition to prioritizing the expansion of health insurance coverage and reducing out-of-pocket health expenditures, a key pillar toward achieving UHC is the strengthening of the country’s primary health care (PHC) system (1) and promotion of the efficient use of existing PHC resources (2, 3).

Under the PHC Strategic Framework (2019–2024), the GoK introduced a comprehensive package of PHC services to be provided among its network of public service providers in all 47 counties (2). The Kenya PHC package consists of 309 interventions encompassing promotive, preventive, ambulatory, and emergency response services in addition to acute ambulatory care, selective chronic disease care, palliative care, and rehabilitative care (Supplementary 1). The strategy also specifies facility standards for appropriate staffing, commodities, equipment, and infrastructure.

Building upon this strategy, the GoK’s Primary Care Network (PCN) guidelines aim to improve the overall operational efficiency of primary care service delivery at public facilities (3, 4). Taking the form of a “hub and spoke model,” (Supplementary 2) a sub-county hospital serves as the hub to numerous lower-level health centers (level 3), dispensaries (level 2), and community health volunteers (CHVs), responsible for reaching the most remote and disadvantaged populations (3). Working together as integrated teams, PCNs deliver services to a defined catchment population, ensuring the continuity of care through their referral and counter-referral systems. PCNs are designed to reduce costs through more efficient use of resources – e.g., shifting services from higher to lower-level facilities, referring complicated cases up to hospitals, sharing critical information (e.g., on suspected disease outbreaks), while addressing stockouts through a shared supply of commodities (3–5).

In the face of these reforms, PHC services remain unavailable, inaccessible, or unaffordable for many Kenyans. According to estimates, 52% of the population lives within a five-kilometer radius of a health facility (2), and coverage of essential health services (Sustainable Development Goal Indicator 3.8.1) is estimated at 56% (6). For those who are able to access services, there is considerable variation in the quality of service provision due to issues of staff capacity and a lack of infrastructure and equipment (7). Although user fees were abolished at level 2 and 3 facilities in 2004, patients continue to face unexpected OOP payments – e.g., due to cost-sharing at public hospitals and accessing care through the private sector (8) – with the incidence of catastrophic payments more severe for the poorest and most rural households, and for those seeking outpatient services (9, 10).

Limited government spending combined with existing inefficiencies within the health system, remain key challenges in expanding access to affordable and quality PHC services. Following Kenya’s transition to a devolved government system in 2013, county-level governments are responsible for mobilizing the majority of funding for primary and secondary health service delivery (11, 12) while the national government provides strategic commodities (e.g., vaccines, family planning commodities, HIV and TB medicines) to primary care facilities. Although there have been incremental increases in health budget allocations (4), county-level budgets are largely considered “wishlists” and lack local-level input to inform realistic target setting (13). Moreover, counties report inadequate staff capacity for leading the budgeting process (14), while political interests continue to play a major role in funding allocations (15). Government expenditures account for just 38% of total PHC expenditure followed by private (35%) and external sources (27%) (16). Delays in government funding to health facilities and poor budget absorption rates have only exacerbated these financing challenges (17). Furthermore, the maldistribution of human resources toward higher-level health facilities has contributed to low quality services and led to unnecessary referrals to higher level hospitals (18).

To guide the implementation of these governmental reforms, evidence is needed on current PHC utilization and costs, as well as the expected costs for ensuring universal coverage of high-quality PHC services. Generating evidence based on resource needs, as opposed to historical utilization (which may reflect resource constraints), can facilitate sufficient resource mobilization and inform priority setting (19) by national and county governments as well as development partners. This study sought to estimate the primary care service delivery costs in public sector primary care facilities in six counties. We compared actual costs to normative costs associated with the Kenya PHC package, with the corresponding gap representing the financial resources needed for providing universal coverage of PHC services in public sector facilities. The key research questions that this study sought to answer were: (1) what is the actual cost of delivering PHC services in sampled facilities; (2) what is the normative cost (i.e., what *should* it cost to deliver the PHC package); and (3) what is the estimated financial resource gap for delivering PHC services based on the difference between actual and normative values?

## 2. Materials and methods

### 2.1. Costing tool

We used the publicly available Primary Health Care Costing, Analysis, and Planning (PHC-CAP) Tool (20) to facilitate the calculation of actual and normative costs. The PHC-CAP Tool is an activity-based costing tool based in Microsoft Excel. Actual costs are calculated using data gathered from a sample of health facilities which are extrapolated to the entire universe of facilities in the area of interest. Normative costs represent the resources needed to provide the services with reasonable quality and efficiency (19). Normative costs are estimated using standard treatment protocols (STPs) for each health service and intervention (21) in the PHC package, with the estimated populations in need for each service based on the population size (reported by sub-counties) and expected service need given estimated rate of disease incidence and prevalence as well as utilization rates for promotional and preventive services. The difference between actual and normative costs represents the resource gap. Aligned with the World Health Organization’s (WHO) PHC Measurement Framework and Indicators (22), the PHC-CAP Tool incorporates key metrics including total cost, cost per capita, cost per service and program, cost per input, numbers of inpatient and outpatient services per clinical staff, and average daily service output per clinical staff. In addition to this application in Kenya, the PHC-CAP Tool has been used in five other countries including Ethiopia (23) and Nigeria (24).

### 2.2. Sampling and data collection

We used multi-stage sampling to select the health facilities included in the study. In a first stage, we selected six out of 47 counties in consultation with the Ministry of Health (MOH; Supplementary 3). Of the six counties, three (Nyeri, Kisumu, and Isiolo) were selected due to their participation in the Afya Care UHC pilot which entitles registered households to free services in level 4 and level 5 public hospitals (25), two (Kajiado and Kilifi) were chosen given their geographic diversity (Rift Valley and coastal, respectively), and one (Makueni) was selected for its health financing scheme “Makueni Care” which provides free care for registered users at public hospitals (26). In a second stage, we selected seven sub-counties based on the following criteria: (a) presence of a referral hospital and (b) outpatient per capita utilization rate was within the median range for that sub-county based on 2018–2019 data in the Kenya Health Information System (KHIS). One sub-county was selected in each county except in Nyeri where two were selected.

Within each sub-county, data were collected from each sub-county hospital and a sample of health centers, dispensaries, and community health units (CHUs) attached to each facility. The sample of health centers was based on total outpatient utilization – i.e., with preference for those with higher outpatient utilization. Dispensaries for which the total outpatient utilization figures were closest to the 25% quartile, 50% quartile, and 75% quartile of the overall distribution were selected. No sampling was conducted for CHUs, as their services and costs were captured at the facility level. All facilities sampled were public except for the faith-based Mary Immaculate Hospital which was the only referral hospital in Kieni West. The sampled facilities comprised 38 dispensaries, 26 health centers, seven sub-county hospitals, and associated CHUs (Table 1). Although our aim was to sample (on average) 10 health facilities in each of the seven sub-counties, there was some variation. In some sub-counties (e.g., Kilifi where we sampled six), the number of sampled facilities fell below this aim due to time and travel constraints while in others (Kisumu where we sampled 16), we were able to sample more facilities.

**Table 1:** Sample of health facilities by geographic location.

County	Sub-county	Total population in sub-county	Dispensary	Health center	Primary hospital
Isiolo	Garbatulla	99,729	7	3	1
Kajiado	Kajiado East	191,241	6	3	1
Kilifi	Ganze	191,685	4	2	1
Kisumu	Kisumu West	151,798	3	9	1
Makueni	Kibwezi West	183,639	6	3	1
Nyeri	Kieni West	93,109	6	3	1
Nyeri	Mukurweini	98,532	6	3	1
	Total		38	26	7

Actual costs, irrespective of funding source, reported by health facilities (July 2018–June 2019) included recurrent health facility operating costs (e.g., electricity, water), human resources (i.e., staff salaries of clinical and non-clinical personnel), and other recurrent costs. Drug and medical supply costs were reported through the Kenya Medical Supplies Authority (KEMSA) database. Data on facility expenditures were reported directly from facilities using checklists and semi-structured interviews; catchment population sizes and budgets were reported through county and sub-county annual work plans. Data on capital costs and above-facility costs (e.g., national and county support and supervision) were excluded from this analysis. The study was conducted from the public sector perspective and did not set out to capture patient costs, including out-of-pocket expenditures on drugs and medical supplies. Data collection was conducted from February to June 2020. The currency exchange rate used was 1 US dollar (USD) equal to 101.99 Kenyan schilling (Ksh) to allow for international comparison (27).

We collected data on the number of inpatient and outpatient services for each facility and sub-county from the KHIS system. Only services reported from July 2018 to June 2019 were included in this analysis to ensure consistency with the cost analysis. Service data were reported monthly and categorized according to KHIS service classifications. For example, the KHIS reported the number of family planning attendances by facility but did not report the disaggregated quantity of individuals receiving oral contraceptives, condoms (male or female), or injectable contraceptives. Although most sub-county hospitals and health centers reported numbers of inpatient cases (for persons younger and older than 5 years old), only five of seven sub-county hospitals reported data on the case mix of inpatient services. Moreover, PHC services provided by CHUs were not separated from facility-based services.

### 2.3. Costing approach

We calculated total annual PHC costs for each facility in the sample. Costs of clinical labor, drugs, medical supplies, and other indirect costs were summed and allocated to outpatient visits and inpatient days based on weighted service volumes in dispensaries, health centers, and sub-county hospitals using WHO-CHOICE data to create the relative cost of an outpatient visit compared to an inpatient day (28). This same WHO-CHOICE ratio of inpatient to outpatient costs was also used to estimate weighted daily caseloads of clinical staff: a ratio of 432:126 in primary hospitals, 432:111 in health centers, and 432:90 in dispensaries (28). In the absence of reliable time use data by service type, this method (i.e., using WHO-CHOICE ratios) was particularly useful at higher level facilities (health centers and hospitals) which provided both inpatient and outpatient services, allowing us to allocate costs according to their estimated resource use.

To estimate the actual total cost per capita of PHC in the seven sub-counties beyond the sampled facilities to the universe of facilities (i.e., extrapolation), we used the following three-step approach (29). First, for each of the facility types (dispensary, health center, and hospital), we compared the total number of outpatient services in our sample with the number of outpatient services in the universe of facilities (also obtained from the KHIS system), and inflated our sample costs in proportion. This follows the assumption that our sample is representative of the sub-counties, and Kenya in general, in terms of outpatient service unit cost. Second, we added the estimates for dispensaries, health centers, and hospitals within a given sub-county, and Kenya in general. Lastly, the actual cost per capita was calculated by dividing the total actual cost by the reported total sub-county or Kenyan population.

Normative costs were estimated based on STPs which were developed for clinical services included in the PHC service package, excluding those not provided at health facilities.^1^ STPs were developed for approximately 24% (73 of 309) services in the PHC package, corresponding to more than 80% of all services provided. Each outpatient service from the Kenya PHC package of services was consolidated and grouped by service-related disease category (i.e., matched) according to the corresponding KHIS service grouping classification. Each service group category received proportional weight in its contribution to the overall cost per KHIS groupings based on previous frequency of use. Normative costs per KHIS grouping were calculated and reported for each level of the PHC network (hospital, health center, and dispensary).

For each STP, information was gathered on the following: total drug, diagnostic, and laboratory reagent requirements per service episode and the corresponding unit cost for each; average number of annual encounters per service episode; total human-resource requirements, based on the number of minutes required per service by human resource cadre and the corresponding salary cost; population in need, computed based on the population of each sub-county and disease incidence and prevalence rates for each service sourced from either national data or from the 2019 Global Burden of Disease (GBD) Kenya dataset (30). Each STP was developed and reviewed by members of an expert panel comprised of MOH staff representing various departments^2^ with a subsequent review conducted by a primary care physician.

We assumed that 30% of clinical health worker time was dedicated to necessary non-clinical activities (e.g., administration and reporting, training, and time spent between consultations) which we considered a conservative estimate for the amount of non-clinical work necessary to provide quality PHC services for the entirety of the catchment population. To account for indirect costs not considered in the STPs, such as non-clinical personnel, utilities and other operational expenses, an overhead rate was calculated based on our sampled facilities, and added to the normative human resource, drug, and medical supply costs. The overhead rate for each facility level was calculated by dividing total indirect cost by the sum of clinical labor, drug and medical supply costs, and indirect costs. Using this approach, we projected the normative costs for public sector PHC facilities to deliver high-quality care for an expected service volume which reflects all needed services among catchment populations in the specified geographic area. This projection, aiming at UHC, assumes that all (100%) needed PHC services are met. Although aspirational, this scenario is indicative of the maximum service coverage expected.

### 2.4. Sensitivity analysis

We conducted one-way sensitivity analyses to understand the impact of various factors on the actual and normative costs per capita and displayed them in tornado diagrams. For both the actual and normative cost per capita, we conducted the same variable adjustments and also estimated best- and worst-case scenarios by varying all of these factors together. We varied clinical labor costs by ±20% given the potential uncertainty of facility reported salaries, the reported problem of ghost workers on government payroll (and potential to curb such costs) (18), and future plans to remunerate CHVs. We varied drug costs by ±30% and supply costs by ±20% to account for any data inaccuracies in KEMSA. We varied indirect costs by ±30% since the indirect costs incorporated in the normative estimates were derived from our actual costs. We also varied population by ±5% given the uncertainty of the figures reported in annual workplans.

## 3. Results

### 3.1. Sample facility characteristics

Health facility staffing patterns within the sample varied by sub-county and by facility level (Table 2). Sub-county hospitals had an average (mean) of 100 total staff (ranging from 43 in Garbatulla to 175 in Kibwezi West) with an average of 65 clinical staff (range 21–143). Among the 26 sampled health centers, there were an average of 36 total staff (range 2–109), with an average of 30 clinical staff (range 2–92) with some health centers reporting no non-clinical staff support.^3^ Among the 38 sampled dispensaries, there were an average of five total staff (range 3–20) and three clinical staff (range 1–17).

**Table 2:** Characteristics of sampled facilities.

County	Sub-county	Facility level	N	Total number of staff		Total number of clinical staff		Total number of non-clinical staff		Total outpatient visits		Total inpatient bed days		Number of OP visits per clinical staff per day		Number of weighted services per clinical staff per day	
				Mean	Range	Mean	Range	Mean	Range	Mean	Range	Mean	Range	Mean	Range	Mean	Range
Isiolo	Garbatulla	Dispensary	7	4.6	3.0–6.0	2.1	1.0–3.0	2.4	2.0–4.0	4,542	2,355–8,964	22	0–154	8.0	4.3–13.6	8.2	4.3–13.6
Isiolo	Garbatulla	Health center	3	26.0	8.0–57.0	21.0	3.0–52.0	5.0	5.0–5.0	11,247	5,974–16,063	277	162–375	4.7	1.2–7.5	5.2	1.2–8.2
Isiolo	Garbatulla	Primary hospital	1	43.0	43.0–43.0	21.0	21.0–21.0	22.0	22.0–22.0	9,620	9,620–9,620	666	666–666	1.7	1.7–1.7	2.1	2.1–2.1
Kajiado	Kajiado East	Dispensary	6	7.2	3.0–20.0	4.7	1.0–17.0	2.5	2.0–4.0	12,432	4,336–43,294	0	0–0	13.5	7.5–22.4	13.5	7.5–22.4
Kajiado	Kajiado East	Health center	3	83.0	42.0–109.0	73.0	36.0–92.0	10.0	6.0–18.0	30,123	14,929–54,511	1	0–4	1.5	0.9–2.2	1.5	0.9–2.2
Kajiado	Kajiado East	Primary hospital ^a^	1	169.0	169.0–169.0	119.0	119.0–119.0	50.0	50.0–50.0	101,672	101,672–101,672	0	0–0	3.2	3.2–3.2	3.2	3.2–3.2
Kilifi	Ganze	Dispensary	4	5.5	3.0–10.0	3.3	2.0–6.0	2.3	0.0–4.0	16,421	10,551–23,229	0	0–0	21.9	13.2–30.1	21.9	13.2–30.1
Kilifi	Ganze	Health center	2	62.5	44.0–81.0	54.5	37.0–72.0	8.0	7.0–9.0	30,522	17,732–43,312	1	0–2	2.0	1.8–2.3	2.0	1.8–2.3
Kilifi	Ganze	Primary hospital	1	64.0	64.0–64.0	40.0	40.0–40.0	24.0	24.0–24.0	54,507	54,507–54,507	1,373	1,373–1,373	5.1	5.1–5.1	5.5	5.5–5.5
Kisumu	Kisumu West	Dispensary	3	7.0	6.0–8.0	5.3	4.0–7.0	1.7	0.0–3.0	12,526	7,809–17,488	0	0–0	8.9	5.8–11.5	8.9	5.8–11.5
Kisumu	Kisumu West	Health center	9	13.9	2.0–46.0	10.9	2.0–39.0	3.0	0.0–7.0	10,662	3,418–23,061	1	0–8	5.5	1.7–11.1	5.5	1.7–11.1
Kisumu	Kisumu West	Primary hospital	1	66.0	66.0–66.0	40.0	40.0–40.0	26.0	26.0–26.0	26,178	26,178–26,178	14,507	14,507–14,507	2.5	2.5–2.5	7.1	7.1–7.1
Makueni	Kibwezi West	Dispensary	6	3.7	3.0–5.0	1.2	1.0–2.0	2.5	2.0–3.0	7,571	5,260–12,320	0	0–0	25.8	15.2–46.1	25.8	15.2–46.1
Makueni	Kibwezi West	Health center	3	34.0	31.0–40.0	28.7	25.0–36.0	5.3	4.0–6.0	22,101	13,504–34,834	3	0–8	3.1	1.4–5.2	3.1	1.4–5.2
Makueni	Kibwezi West	Primary hospital	1	175.0	175.0–175.0	143.0	143.0–143.0	32.0	32.0–32.0	106,352	106,352–106,352	2,001	2,001–2,001	2.8	2.8–2.8	3.0	3.0–3.0
Nyeri	Kieni West	Dispensary	6	4.5	3.0–6.0	2.3	1.0–3.0	2.2	2.0–3.0	9,007	5,256–16,516	0	0–0	16.4	7.2–34.5	16.4	7.2–34.5
Nyeri	Kieni West	Health center	3	53.0	36.0–72.0	46.0	30.0–65.0	7.0	6.0–8.0	25,530	19,969–29,261	10	0–30	2.2	1.6–2.5	2.2	1.6–2.5
Nyeri	Kieni West	Primary hospital	1	74.0	74.0–74.0	29.0	29.0–29.0	45.0	45.0–45.0	18,997	18,997–18,997	8,641	8,641–8,641	2.5	2.5–2.5	6.3	6.3–6.3
Nyeri	Mukurweini	Dispensary	6	5.2	3.0–10.0	2.5	2.0–4.0	2.7	1.0–6.0	12,616	7,022–20,873	0	0–0	19.0	13.1–24.6	19.0	13.1–24.6
Nyeri	Mukurweini	Health center	3	30.0	20.0–37.0	24.7	16.0–30.0	5.3	4.0–7.0	18,969	14,175–21,790	10	0–30	2.9	2.7–3.3	3.0	2.7–3.3
Nyeri	Mukurweini	Primary hospital	1	115.0	115.0–115.0	65.0	65.0–65.0	50.0	50.0–50.0	56,205	56,205–56,205	19,801	19,801–19,801	3.2	3.2–3.2	7.2	7.2–7.2
	Total	Dispensary	38	5.2	3.0–20.0	2.8	1.0–17.0	2.4	0.0–6.0	10,127	2,355–43,294	4	0–154	16.3	4.3–46.1	16.3	4.3–46.1
	Total	Health center	26	35.7	2.0–109.0	30.3	2.0–92.0	5.4	0.0–18.0	18,497	3,418–54,511	35	0–375	3.7	0.9–11.1	3.8	0.9–11.1
	Total	Primary hospital	7	100.9	43.0–175.0	65.3	21.0–143.0	35.6	22.0–50.0	53,362	9,620–106,352	6,713	0–19,801	3.0	1.7–5.1	4.9	2.1–7.2

^a^The sub-county hospital in Kajiado East did not report inpatient bed days.

Among the sampled facilities, hospitals accounted for the highest per facility volume of outpatient services and inpatient bed days, with fewer inpatient services delivered at the health center levels. Among the different facility types, there was a considerable range of total outpatient services delivered annually – e.g., in Garbatulla, one dispensary only provided 2,355 services while a dispensary in Kajiado East provided more than 43,000 services. In terms of the total volume of outpatient services delivered within the sampled sub-counties (Table 3), dispensaries provided the majority of services (46.23%), followed by health centers (29.74%), and hospitals (24.03%). This contrasted slightly with national-level service data reported from the KHIS for dispensaries (40.8%), health centers (25.6%), and hospitals (33.7%).

**Table 3:** Percentage of outpatient services delivered by type of health facility (2018–2019).

County	Sub-county	Dispensaries	Health centers	Primary hospitals
		(% services)	(% services)	(% services)
Isiolo	Garbatulla	51.7%	37.6%	10.7%
Kajiado	Kajiado East	37.3%	29.5%	33.2%
Kilifi	Ganze	60.2%	21.0%	18.8%
Kisumu	Kisumu West	22.0%	41.9%	36.1%
Makueni	Kibwezi West	53.3%	22.9%	23.8%
Nyeri	Kieni West	40.3%	49.6%	10.1%
Nyeri	Mukurweini	57.0%	21.6%	21.4%
	Average of 7 sub-counties	46.2%	29.7%	24.0%
	National^a^	40.8%	25.6%	33.7%

^a^Based on KHIS data for the universe of facilities.

The daily caseload of clinical staff (also shown in Table 2) highlights the variations in staffing patterns and service output. On average, clinical staff provided 3.0 outpatient services per day at hospitals, 3.7 at health centers, and 16.3 services per day at dispensaries. When weighing both outpatient and inpatient services according to WHO-CHOICE ratios, hospitals accounted for more daily caseloads per clinical staff. Due to data availability, a more in depth analysis of outpatient case mix was not possible as it would have required detailed data from health facility registries. Moreover, only six sampled hospitals reported inpatient data and only five hospitals recorded inpatient case mix, therefore a complete analysis of inpatient service delivery was not possible.

### 3.2. Actual and normative costs in the sample and network of facilities

**Figure 1:**
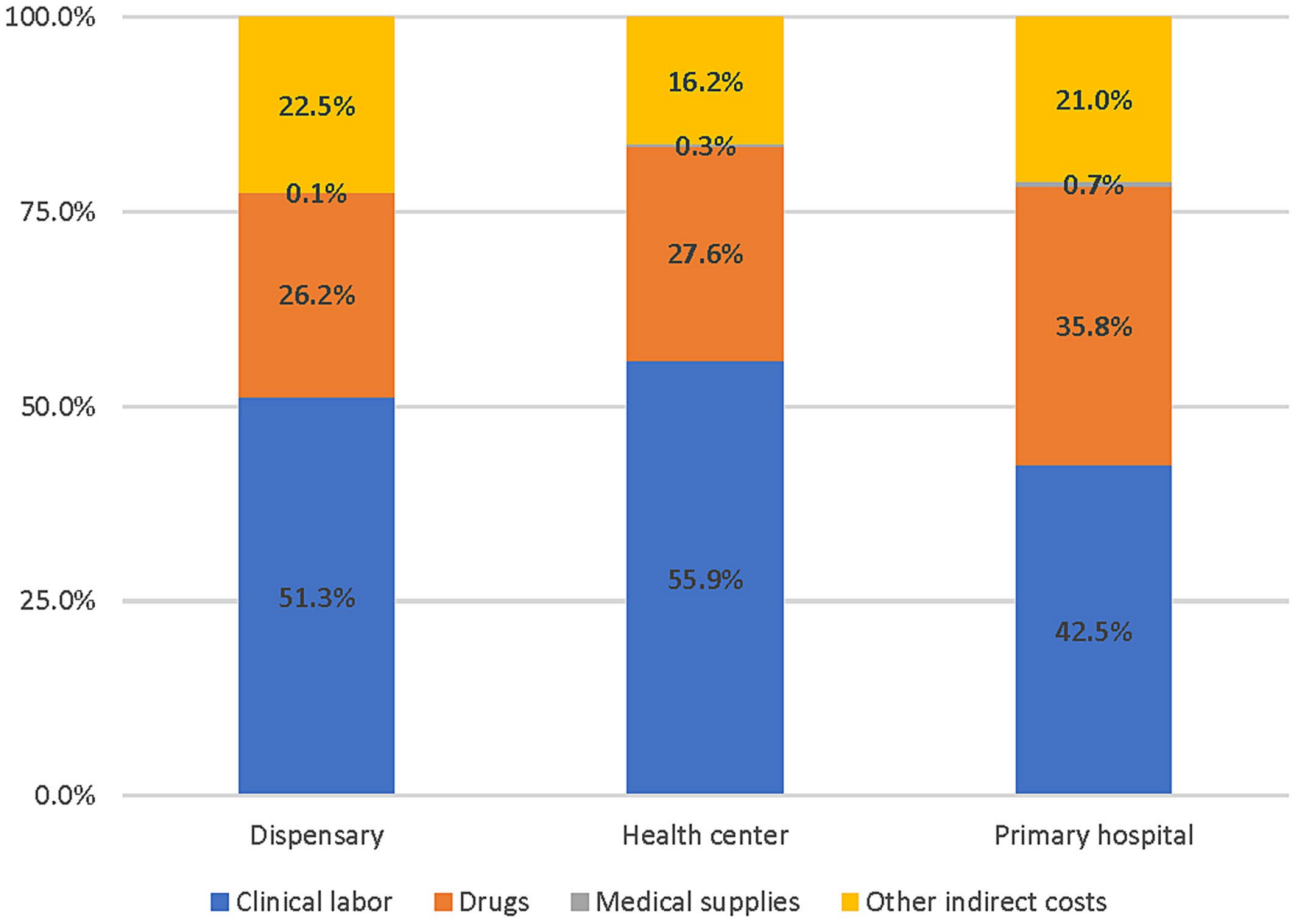
Distribution of PHC costs by cost categories and facility type (2018–2019).

Among the facilities sampled, average annual costs of delivering PHC services varied considerably across facility types and the sub-counties (Table 4): dispensaries (US$ 12,794 – US$ 237,486); health centers (US$ 30,235 – US$ 1.5 M); and hospitals (US$ 498,674 – US$ 5.5 M). Clinical labor costs represented the largest cost driver among all facility types, followed by drugs, indirect costs, and medical supplies (Figure 1).

**Table 4:** Distribution of total costs in sampled facilities.

**County**	**Sub-county**	**Facility level**	**N**	**Clinical labor (USD)**		**Drugs (USD)**		**Medical supplies (USD)**		**Other indirect costs (USD)**		**Total costs (USD)**	
				**Mean**	**Range**	**Mean**	**Range**	**Mean**	**Range**	**Mean**	**Range**	**Mean**	**Range**
Isiolo	Garbatulla	Dispensary	7	20,573	5,530–28,591	13,528	9,430–21,068	0	0–0	4,451	706–19,767	38,553	15,703–51,443
				52.2%	32.7%–73.4%	37.5%	23.1%–60.3%	0.0%	0.0%–0.0%	10.3%	2.2%–44.3%	100.0%	100.0%–100.0%
Isiolo	Garbatulla	Health center	3	78,341	23,591–150,015	32,713	13,362–44,429	20	0–59	14,736	13,295–16,708	125,809	50,306–211,152
				57.0%	46.9%–71.0%	27.5%	21.0%–34.8%	0.0%	0.0%–0.1%	15.5%	7.9%–26.4%	100.0%	100.0%–100.0%
Isiolo	Garbatulla	Primary hospital	1	302,853	302,853–302,853	98,987	98,987–98,987	0	0–0	96,833	96,833–96,833	498,674	498,674–498,674
				60.7%	60.7%–60.7%	19.9%	19.9%–19.9%	0.0%	0.0%–0.0%	19.4%	19.4%–19.4%	100.0%	100.0%–100.0%
Kajiado	Kajiado East	Dispensary	6	41,847	882–137,543	16,068	5,232–48,745	222	0–923	17,893	8,285–50,275	76,031	14,399–237,486
				39.2%	3.9%–69.4%	27.7%	14.2%–53.7%	0.2%	0.0%–0.6%	32.9%	15.8%–57.5%	100.0%	100.0%–100.0%
Kajiado	Kajiado East	Health center	3	206,267	91,950–315,772	378,469	29,365–1,074,441	977	275–1,696	63,446	24,524–124,041	649,160	146,113–1,515,950
				52.6%	20.8%–74.0%	34.0%	11.1%–70.9%	0.2%	0.1%–0.3%	13.2%	8.2%–16.8%	100.0%	100.0%–100.0%
Kajiado	Kajiado East	Primary hospital	1	1,695,249	1,695,249–1,695,249	3,485,608	3,485,608–3,485,608	21,473	21,473–21,473	377,706	377,706–377,706	5,580,035	5,580,035–5,580,035
				30.4%	30.4%–30.4%	62.5%	62.5%–62.5%	0.4%	0.4%–0.4%	6.8%	6.8%–6.8%	100.0%	100.0%–100.0%
Kilifi	Ganze	Dispensary	4	31,100	20,560–53,619	27,429	11,079–59,983	77	0–237	8,701	0–16,489	67,306	39,952–130,139
				49.6%	40.5%–72.3%	37.9%	27.7%–46.1%	0.1%	0.0%–0.5%	12.4%	0.0%–18.9%	100.0%	100.0%–100.0%
Kilifi	Ganze	Health center	2	107,931	74,218–141,643	63,632	32,399–94,866	3,251	2,474–4,029	45,323	37,569–53,078	220,137	148,214–292,061
				49.3%	48.5%–50.1%	27.2%	21.9%–32.5%	1.8%	0.8%–2.7%	21.8%	18.2%–25.3%	100.0%	100.0%–100.0%
Kilifi	Ganze	Primary hospital	1	355,667	355,667–355,667	126,676	126,676–126,676	701	701–701	135,420	135,420–135,420	618,464	618,464–618,464
				57.5%	57.5%–57.5%	20.5%	20.5%–20.5%	0.1%	0.1%–0.1%	21.9%	21.9%–21.9%	100.0%	100.0%–100.0%
Kisumu	Kisumu West	Dispensary	3	46,909	23,085–81,813	21,290	20,412–22,537	39	0–118	9,352	5,773–14,215	77,590	49,388–118,565
				57.0%	46.7%–69.0%	30.9%	19.0%–41.3%	0.1%	0.0%–0.2%	12.0%	11.7%–12.4%	100.0%	100.0%–100.0%
Kisumu	Kisumu West	Health center	9	50,372	18,943–138,158	26,612	8,347–48,942	218	0–935	15,654	2,910–41,305	92,857	30,235–216,597
				53.6%	29.8%–66.1%	30.4%	22.1%–44.2%	0.2%	0.0%–0.6%	15.8%	5.8%–25.9%	100.0%	100.0%–100.0%
Kisumu	Kisumu West	Primary hospital	1	340,622	340,622–340,622	221,444	221,444–221,444	11,708	11,708–11,708	167,808	167,808–167,808	741,581	741,581–741,581
				45.9%	45.9%–45.9%	29.9%	29.9%–29.9%	1.6%	1.6%–1.6%	22.6%	22.6%–22.6%	100.0%	100.0%–100.0%
Makueni	Kibwezi West	Dispensary	6	9,277	4,706–18,157	3,979	2,479–5,539	0	0–0	9,836	5,609–12,235	23,093	12,794–33,835
				37.9%	23.1%–53.7%	18.2%	10.2%–24.7%	0.0%	0.0%–0.0%	44.0%	33.5%–60.1%	100.0%	100.0%–100.0%
Makueni	Kibwezi West	Health center	3	92,701	41,553–158,691	64,236	8,125–169,414	175	0–524	23,767	18,978–30,688	180,878	78,357–358,792
				57.0%	44.2%–73.8%	24.8%	7.7%–47.2%	0.2%	0.0%–0.5%	18.1%	8.6%–27.6%	100.0%	100.0%–100.0%
Makueni	Kibwezi West	Primary hospital	1	2,240,773	2,240,773–2,240,773	1,110,291	1,110,291–1,110,291	65,171	65,171–65,171	791,899	791,899–791,899	4,208,134	4,208,134–4,208,134
				53.2%	53.2%–53.2%	26.4%	26.4%–26.4%	1.5%	1.5%–1.5%	18.8%	18.8%–18.8%	100.0%	100.0%–100.0%
Nyeri	Kieni West	Dispensary	6	26,879	14,160–38,261	7,846	3,429–11,047	105	0–632	7,103	3,643–14,173	41,933	28,385–58,885
				63.1%	48.9%–79.1%	18.8%	12.1%–28.1%	0.2%	0.0%–1.1%	17.9%	7.5%–29.2%	100.0%	100.0%–100.0%
Nyeri	Kieni West	Health center	3	145,994	102,388–171,446	238,753	16,607–681,436	736	196–1,228	43,748	20,487–57,175	429,232	139,677–903,545
				53.9%	18.2%–73.3%	31.6%	7.5%–75.4%	0.2%	0.1%–0.5%	14.3%	6.3%–21.9%	100.0%	100.0%–100.0%
Nyeri	Kieni West	Primary hospital	1	152,915	152,915–152,915	266,073	266,073–266,073	5,569	5,569–5,569	251,674	251,674–251,674	676,230	676,230–676,230
				22.6%	22.6%–22.6%	39.3%	39.3%–39.3%	0.8%	0.8%–0.8%	37.2%	37.2%–37.2%	100.0%	100.0%–100.0%
Nyeri	Mukurweini	Dispensary	6	27,348	20,282–40,803	7,965	4,452–17,556	14	0–83	10,762	4,859–27,567	46,089	30,487–85,926
				62.1%	47.5%–67.5%	16.8%	14.0%–20.4%	0.0%	0.0%–0.2%	21.1%	13.5%–32.1%	100.0%	100.0%–100.0%
Nyeri	Mukurweini	Health center	3	77,585	59,203–93,356	12,781	10,292–14,950	177	0–485	19,706	12,214–26,144	110,250	81,754–134,450
				70.6%	69.4%–72.4%	11.7%	11.1%–12.6%	0.2%	0.0%–0.4%	17.5%	14.9%–19.4%	100.0%	100.0%–100.0%
Nyeri	Mukurweini	Primary hospital	1	1,020,479	1,020,479–1,020,479	1,970,513	1,970,513–1,970,513	18,099	18,099–18,099	765,596	765,596–765,596	3,774,687	3,774,687–3,774,687
				27.0%	27.0%–27.0%	52.2%	52.2%–52.2%	0.5%	0.5%–0.5%	20.3%	20.3%–20.3%	100.0%	100.0%–100.0%
	Total	Dispensary	38	27,401	882–137,543	12,722	2,479–59,983	65	0–923	9,673	0–50,275	49,862	12,794–237,486
				51.3%	3.9%–79.1%	26.2%	10.2%–60.3%	0.1%	0.0%–1.1%	22.5%	0.0%–60.1%	100.0%	100.0%–100.0%
	Total	Health center	26	95,072	18,943–315,772	97,986	8,125–1,074,441	566	0–4,029	27,990	2,910–124,041	221,614	30,235–1,515,950
				55.9%	18.2%–74.0%	27.6%	7.5%–75.4%	0.3%	0.0%–2.7%	16.2%	5.8%–27.6%	100.0%	100.0%–100.0%
	Total	Primary hospital	7	872,651	152,915–2,240,773	1,039,942	98,987–3,485,608	17,531	0–65,171	369,562	96,833–791,899	2,299,686	498,674–5,580,035
				42.5%	22.6%–60.7%	35.8%	19.9%–62.5%	0.7%	0.0%–1.6%	21.0%	6.8%–37.2%	100.0%	100.0%–100.0%

*Indirect costs include non-clinical and support staff, training & social mobilization, utilities & communications, other recurrent costs and capital/equipment maintenance.

Based on the extrapolation of sampled facility costs to the universe of facilities, the total delivery cost in the seven sub-counties ranged from $US 1.3 million in Garbatulla to US$ 9.3 million in Kajiado East (Table 5). Based on reported sub-county populations, this corresponds to a per capita cost ranging from US$ 9.30 in Ganze to US$ 47.2 in Mukurweini. The total annual PHC delivery costs in Kenya were estimated at $US 1.36 billion (US$ 26.6 per capita) when the sample was extrapolated to all dispensaries, health centers, and sub-county hospitals using KHIS service data. Hospitals made up the vast majority of costs (US$ 948 million).

**Table 5:** Summary of PHC network costs by sub-county (2018–2019).

County	Sub-county	Facility type	N	Total cost in sample (USD)	% Services captured by sample	Extrapolated total cost (USD)	Total delivery cost, whole PHC network (USD)	Cost per capita (USD)
Isiolo	Garbatulla	Dispensary	7	269,873	69%	393,498	1,269,600	12.7
	Garbatulla	Health Center	3	377,428	100%	377,428		
	Garbatulla	Primary hospital	1	498,674	100%	498,674		
Kajiado	Kajiado East	Dispensary	6	456,184	65%	700,052	8,227,566	43.0
	Kajiado East	Health Center	3	1,947,479	100%	1,947,479		
	Kajiado East	Primary hospital	1	5,580,035	100%	5,580,035		
Kilifi	Ganze	Dispensary	4	269,225	38%	717,690	1,776,429	9.3
	Ganze	Health Center	2	440,275	100%	440,275		
	Ganze	Primary hospital	1	618,464	100%	618,464		
Kisumu	Kisumu West	Dispensary	3	232,770	62%	376,642	4,206,786	27.7
	Kisumu West	Health Center	9	835,710	83%	1,007,191		
	Kisumu West	Primary hospital	1	741,581	26%	2,822,954		
Makueni	Kibwezi West	Dispensary	6	138,555	19%	724,814	5,768,441	31.4
	Kibwezi West	Health Center	3	542,635	65%	835,494		
	Kibwezi West	Primary hospital	1	4,208,134	100%	4,208,134		
Nyeri	Kieni West	Dispensary	6	251,597	71%	353,741	2,602,492	28.0
	Kieni West	Health Center	3	1,287,695	82%	1,572,521		
	Kieni West	Primary hospital	1	676,230	100%	676,230		
Nyeri	Mukurweini	Dispensary	6	276,534	51%	547,465	4,652,900	47.2
	Mukurweini	Health Center	3	330,749	100%	330,749		
	Mukurweini	Primary hospital	1	3,774,687	100%	3,774,687		
	Kenya (MOH)	Dispensary	38	1,894,738	1%	175,351,168	1,367,753,095	26.6
National	Kenya (MOH)	Health Center	26	5,761,970	2%	243,666,039		
	Kenya (MOH)	Primary hospital	7	16,097,804	2%	948,735,888		

*Indirect costs include non-clinical and support staff, training and social mobilization, utilities & communications, other recurrent costs, and capital/equipment maintenance.

Table 6 compares the annual actual per capita PHC cost in the seven sub-counties with the normative cost per capita of delivering the Kenya PHC package at universal coverage. The national actual cost per capita (US$ 26.6) was substantially lower than the normative cost per capita (US$ 41.7), which corresponds to a sizeable resource gap of US$ 15.1 per person and the need to increase investment by 1.6 times its current level. The actual cost per capita was lower than the normative cost per capita in all sub-counties with the exception of Mukurweini which suggests there is no financial resource gap in that specific county to achieving universal coverage of the Kenya PHC package.

**Table 6:** Actual and normative costs per capita and required increased investment (2018–2019).

County	Sub-county	Actual cost per capita (USD)	Normative cost per capita (USD)	Required increased investment
Isiolo	Garbatulla	12.7	34.7	2.7 times
Kajiado	Kajiado East	43.0	45.6	1.1 times
Kilifi	Ganze	9.3	31.8	3.4 times
Kisumu	Kisumu West	27.7	36.9	1.3 times
Makueni	Kibwezi West	31.4	42.4	1.4 times
Nyeri	Kieni West	28.0	30.9	1.1 times
Nyeri	Mukurweini	47.2	40.2	0.9 times
National	Kenya (MOH)	26.6	41.7	1.6 times

*These estimates include indirect costs.

### 3.3. Sensitivity analysis

**Figure 2:**
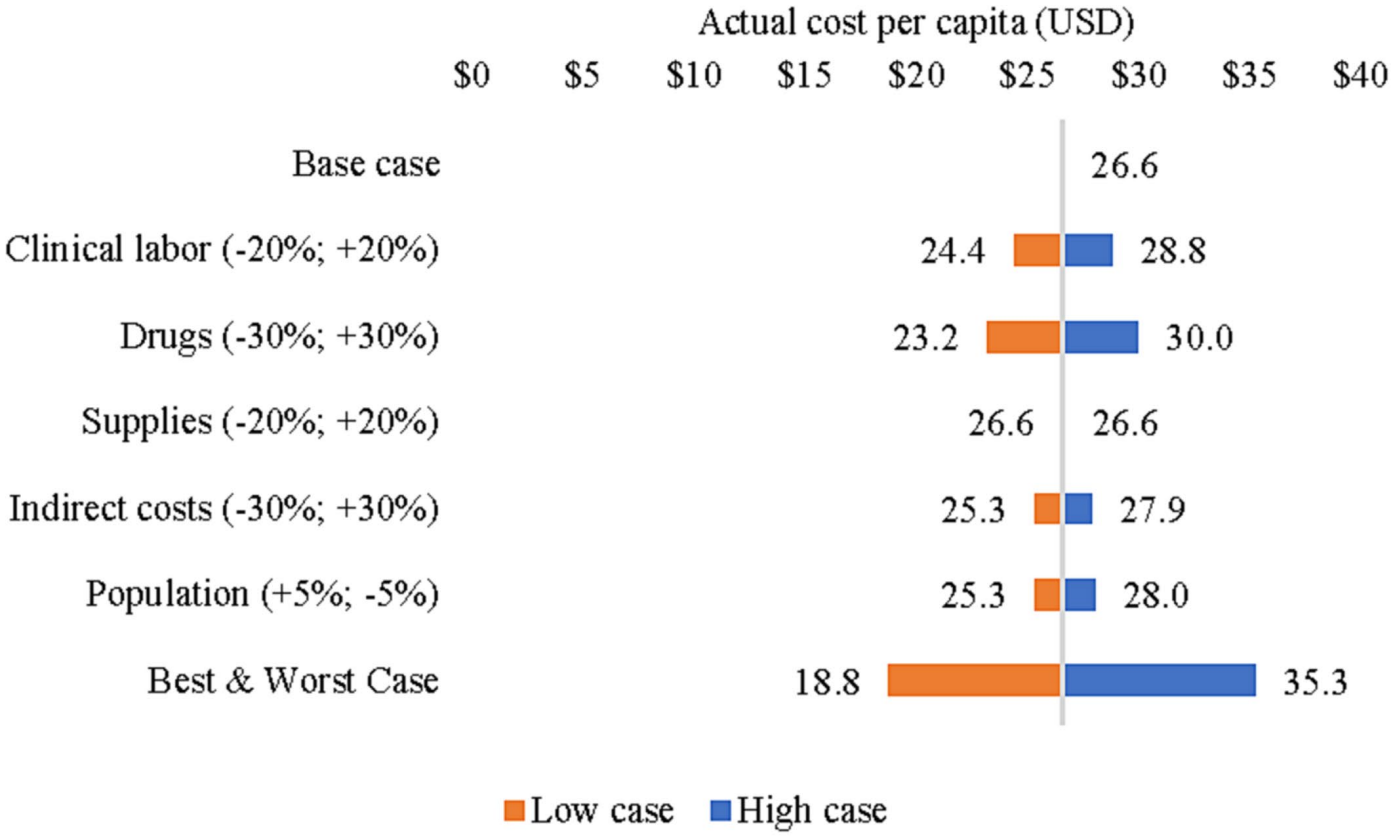
Sensitivity analysis of actual cost per capita (2018–2019).

**Figure 3:**
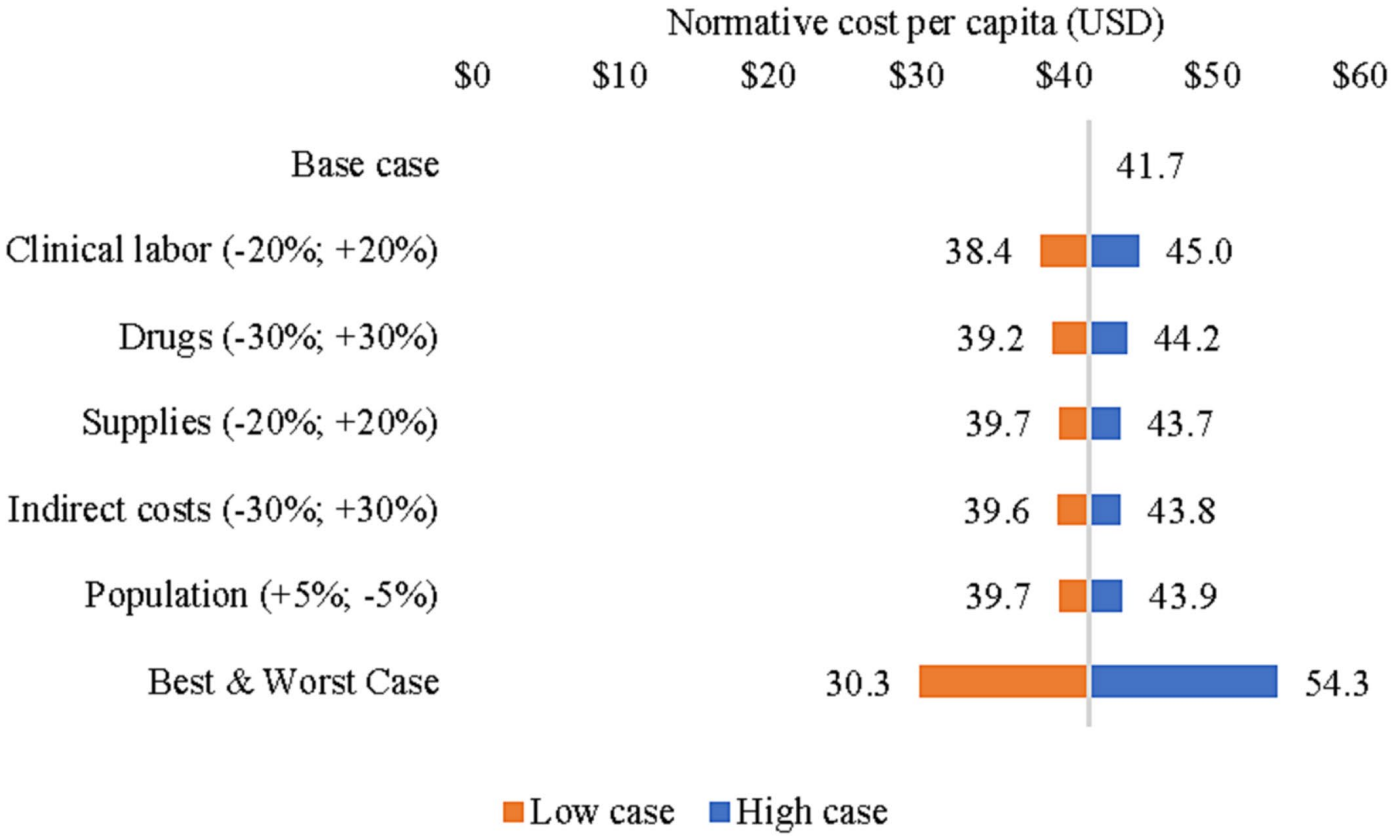
Sensitivity analysis of normative cost per capita (2018–2019).

The sensitivity analyses are presented as tornado diagrams in Figures 1, 2. When clinical labor costs, drug costs, supplies costs, indirect costs, and population size were changed simultaneously to create best- and worst-case scenarios, the resulting actual cost per capita ranged from US$ 18.8 to US$ 35.3 (Figure 2). Meanwhile, the normative cost per capita ranged from US$ 30.3 to US$ 54.3 (Figure 3). Normative costs, which were estimated with other assumptions of necessary non-clinical time, are available in Supplementary 4.

## 4. Discussion

Our study found that the average actual PHC cost per capita ranged from US$ 9.3 in Ganze to US$ 47.2 in Mukurweini while the normative cost per capita ranged from US$ 31.8 in Ganze to US$ 42.4 in Kibwezi West. With the exception of Mukurweini, the results suggest that there is a need for additional investment to ensure universal coverage of Kenya’s PHC package. To our knowledge, only one other relevant peer-reviewed study on PHC costs has been conducted in Kenya which sought to estimate the investment required for operationalizing a PCN in Kenya over a five-year period (4). Although the objectives and ingredients of the costing were different (e.g., it included infrastructure and other capital costs), that study found human resources to be the main cost driver of PCN implementation, accounting for 75% of the total cost. It also found that shifting services from high-level facilities to lower-level PHC facilities could generate significant cost savings.

Comparing the findings of this study to other PHC costing studies from low- and middle-income countries would be misleading given considerable differences in the PHC service packages and heterogeneity of countries (i.e., differences in population, health status and care seeking, gross domestic product, health infrastructure). Nevertheless, studies from Afghanistan (31), Ethiopia (23), and Nigeria (24) have also compared the actual and normative costs of PHC service packages. In line with our study, there is a considerable resource gap between current PHC resources and what is needed to ensure universal coverage of a defined PHC package according to quality standards.

Our study provides granular, facility-level cost estimates for PHC which relied on routine information systems such as the KHIS and the KEMSA database for drug and supply expenditures and data collected from sub-national health offices and facilities. Although the generalizability of the sampled results may be limited, the percentage of services captured by the sample was considerable (Table 5). A major strength of our study was that in four of seven sub-counties, we captured data from all available health centers which accounted for 100% of health-center provided services in these sub-counties. With the exception of Kisumu West (26%), we sampled 100% of all hospitals. As for the normative costs, to our knowledge, no Kenya-specific STPs had been developed prior to this study. Our work could be leveraged to promote clinical practice standards, enhance strategic planning and budgeting in the health sector, and inform provider reimbursements (e.g., from the NHIF). It is also among few studies in low- and middle-income countries that compares the actual and normative PHC costs while estimating the financial resource gap (23, 24, 31).

### 4.1. Policy implications

This study offers valuable evidence on sub-national cost variations and resource requirements to guide the implementation of the GOK’s PHC reforms and resource mobilization efforts. Assuming that the entire Kenyan population would access PHC services in public facilities, our study demonstrates the need for increasing investment by 1.6 times its current level nationally. This suggests that PHC is currently underfunded and would require a substantial increase in financial resources to expand PHC service provision.

Nevertheless, closing the PHC resource gap may also be possible by addressing existing inefficiencies within the health system. For example, our study suggests that there is considerable underutilization of labor and low caseloads among clinical staff in sampled facilities. Low staff utilization among clinical personnel could be due to numerous factors, including, but not limited to staff absenteeism and low demand for services due to geographic or financial barriers or perceived quality of care. This variance in productive efficiency could represent available staff time that could be optimized if the PHC system experienced an increase in service utilization.

When examining labor utilization (i.e., daily services provided per clinical worker), many low-volume facilities spent more financial resources relative to higher-volume facilities, suggesting an inefficiency in labor use. Nevertheless, there are some facilities that report both higher utilization and have relatively lower costs, suggesting that clinical staff spend less time with patients. Our suppositions on inefficiencies within the health facilities are consistent with previous work. A number of previous studies on outpatient services in Kenya have highlighted the inefficiencies that exist in health facilities and that there is variation across facilities (32, 33). Previous work has also highlighted important variation in efficiency across county health systems in Kenya with some counties being quite efficient and others being very inefficient (34).

Furthermore, our study highlights the considerable variation in staffing patterns among health facilities. Although the difference in staffing patterns could be necessary to meet the needs of its catchment populations (especially for facilities in more urban areas requiring more personnel), it could also indicate that health facilities lack clear guidelines for staffing norms. The maldistribution of clinical staff could be due a lack of clear guidance for staffing norms or, as suggested by Nyawira et al. (18), the potentially high prevalence of ghost workers, staff absenteeism, and general over-staffing at higher levels of care (18).

Our findings also suggest there is opportunity to improve the overall efficiency of care by managing referrals through gatekeeping and better informing patients of the referral process (35), as described in the PCN guidelines (3). For example, in Makueni County, patients are required to receive prior authorization from the referral facility before seeking services while providers are on a fee-for-service basis to incentivize high-priority PHC services (36). Similarly, in Nyeri County, the pilot UHC program assigned patients to the nearest health facility where they would initially seek health services so as to reduce overcrowding at sub-country hospitals. The NHIF could also potentially limit reimbursement for certain services provided at hospitals, and strategic purchasing mechanisms (e.g., performance-based financing) could be leveraged to incentivize service provision at specific PHC system levels. Based on KHIS data, hospitals provided more than one-third of the PHC services nationally, indicating there is potential to increase care-seeking at health centers, dispensaries, and CHUs. Although challenging to determine the ideal ratio of service provision across facility levels, there were some categories of services (e.g., diarrhea, malaria, pneumonia) which we would expect to be treated at lower facility levels or CHUs; however, a considerable share are still provided at hospitals.^4^ Unfortunately, the KHIS does not specify the severity of each case, the number of cases with co-morbidities, nor upward and downward referrals.

### 4.2. Limitations

This study did have a number of limitations. The study assumed that the sampled facilities were representative of the PHC network in the seven sub-counties which may limit the expansion of cost results to the universe of facilities and at a national level (for Kenya). Nevertheless, our sampling approach goes beyond many costing studies which commonly use convenience sampling (37). The study also relied on service utilization data from KHIS which in the past has been reportedly known to have quality and completeness issues. Due to aggregation of KHIS categories, it is possible that hospitals also provided secondary services which are not included in the Kenya PHC package, which would have resulted in an overestimate of actual PHC costs. Although national policy prohibits level 2 facilities (dispensaries) from providing inpatient services, our sample indicated a small number of inpatient services were provided. Normative costs were based on standard treatment guidelines and expert panel opinion which are subject to bias. Moreover, individual STPs did not account for potential time savings due to integrated service provision (e.g., simultaneous diagnosis and treatment of multiple conditions). Moreover, Kenya-specific incidence and prevalence estimates for STPs were unavailable for certain conditions and had to be approximated using the best available evidence.

### 4.3. Future research

Additional analysis on staffing mix, task shifting (upstream and downstream), and service outputs (including case mix, quality of service provision, and levels of efficiency) is necessary to inform opportunities for improving PHC efficiency. Further analysis should also be conducted to determine the level of and reasons for bypass, as well as the impact of existing gatekeeping mechanisms. Our analysis also underscores the necessity of improving the nominal classification of health facilities and ensuring adequate human resources are equipped to provide explicit services within the PHC package. Further insight is required into facilities with low service volume per clinical staff (e.g., low demand for services, absenteeism), highlighting the need to closely examine labor patterns and slack capacity to better understand if this is due to idle staff time or absenteeism. Improving the capture of CHV-level data to assess their level of service provision is also critical. Future cost analyses would benefit from more detailed service data within the KHIS (e.g., severity of diagnosis and inpatient case mix data), as well as routinely reported cost and human resource data (number of staff per cadre per facility level). This would reduce the need for sub-county and facility level data collection.

## 5. Conclusion

Amidst Kenya’s ongoing reforms to expand access to high-quality PHC services and improve the overall efficiency of primary care service delivery, our study results show that there is a sizable financial gap between the actual resources for PHC services and the estimated normative costs. In addition to mobilizing additional financial resources for PHC, our study highlights the need to “spend better” and improve the overall efficiency of current PHC expenditures to fully harness the benefits of a high-functioning PHC system and guide the GoK in its vision of achieving UHC.

## Data availability statement

The raw data supporting the conclusions of this article will be made available by the authors, without undue reservation.

## Author contributions

CG and AO contributed to the design of the study. CG, SB, HC, CS, RM, and CU analyzed and interpreted the data, and collected data on costs and service utilization. AO, SH, DN, SM, EW, MO, and DW provided critical revisions and inputs on the relevance of the findings to policy reforms. CG wrote the first draft of the manuscript. All authors reviewed, contributed to the article, and approved the submitted version.

## Footnotes

^1^E.g., food quality and safety monitoring, pollution control, road infrastructure.

^2^These included the following departments: Reproductive and Family Planning Health, Maternal Health, Non-Communicable Diseases, Malaria, HIV/AIDS, Tuberculosis, Neglected Diseases, Immunization, Child Health, Nutrition, Mental Health, Community Health, and the Diagnostic/Clinical Laboratory departments.

^3^Includes non-clinical staff (e.g., technologists, pharmacists, clerks, record keepers) and support staff (e.g., security, cooks, cleaners, grounds person).

^4^In the sampled sub-counties, the following percentage of cases were treated at the hospital level: diarrhea (26%), antenatal care (47%), malaria (16%), pneumonia (31%), acute upper respiratory infections (15%).
