# Supplementary material for: The costs and financing needs of delivering Kenya’s primary health care service package
Source: Front Public Health. 2023 Oct 12;11:1226163. doi: 10.3389/fpubh.2023.1226163 (PMC10613057; doi:10.3389/fpubh.2023.1226163)
Supplement: Supplementary file 1 [file Table_1.docx]

Supplementary Material

The Costs and Financing Needs of Delivering Kenya’s Primary Health Care Service Package

**Agatha Olago, Christian Suharlim, Salim Hussein, David Njuguna, Stephen Macharia, Rodrigo Muñoz, Marjorie Opuni, Hector Castro, Clarisse Uzamukunda, Damian Walker, Sarah Birse, Elizabeth Wangia, Colin Gilmartin***

*** Correspondence:** Colin Gilmartin cgilmartin@msh.org

## Supplementary Figures

# Supplementary material

Supplement 1. Kenya PHC Package of Services (summarized)

The coverage of primary health care services is organized as in the Kenya Essential Package for Health (KEPH) format. Further details on all 309 services can be found in Table of the PHC Strategic Framework.

| **Program** | **Sub Program** |
| --- | --- |
| 1. Family Planning and Reproductive Health | Infertility Management |
|  | Prevention and Treatment of gynecology problems |
|  | Sexual health issues |
|  | Gender Based Violence |
| 2. Maternal, Neonatal and Child Health | Antenatal Care |
|  | Skilled Delivery Care |
|  | Postnatal Care |
|  | Neonatal Care |
|  | Intensive Neonatal Care |
|  | Immunization and Vaccination |
|  | Child Health |
|  | Nutrition |
| 3. Management of HIV/AIDS | Prevention of HIV/AIDS |
|  | HIV/AIDS: Care and Treatment |
| 4. Management of Tuberculosis | TB Screening |
|  | TB Treatment |
| 5. Management of Malaria | Malaria Prevention |
|  | Malaria Case Management |
| 6. Infectious Diseases | Measles |
|  | Mumps |
|  | Pneumonia |
|  | Upper respiratory tract infections |
| 7. Neglected Tropical Diseases | NTD Prevention |
|  | NTD: Leishmaniasis (Kalar Azar) |
|  | NTD: Schistosomiasis |
| 8. Non-Communicable Diseases | Nutritional Disorders |
|  | Cardiovascular |
|  | Ischemic heart disease, stroke, and peripheral artery diseases |
|  | NCD: Respiratory |
|  | NCD: Diabetes mellitus |
|  | NCD: Endocrine |
|  | NCD: Gastrointestinal |
|  | NCD: Genitourinary |
|  | NCD: Cancers |
|  | Colorectal cancer |
|  | Prostate cancer |
|  | Eye conditions |
|  | Skin conditions |
|  | NCD: hematology |
| 9. Surgical, emergency, and critical care | Injury: Emergency |
|  | Injury: Promotion and Prevention |
|  | Injury: Treatment |
|  | Injury: Emergency |
|  | Injury: Psychosocial |
|  | Surgical care: Oral and Dental procedures |
|  | Surgical care: Non-trauma surgical conditions |
| 10. Ear, nose and throat | Ear nose and throat: nose |
|  | Ear nose and throat: eye |
|  | Ear nose and throat |
| 11. Mental, neurological, and substance abuse disorders | Neurological |
|  | Mental health |
|  | Birth defects |
| 12. Rehabilitation | Rehabilitation: speech and hearing |
|  | Rehabilitation: Disabilities |
|  | Rehabilitation: Physiotherapy |
|  | Rehabilitation: Occupational |
|  | Rehabilitation: Psychosocial |
| 13. Collaboration with other sectors | Food quality and safety |
|  | Workplace health and safety |
|  | Port Health: Monitoring |
|  | Multi sector: Water |
|  | Multi sector: WASH |
|  | Multi: Pollution |
|  | Multisector: Housing |
|  | Multi sector: school health |
|  | Multi sector: food fortification |
|  | Multi sector: Population management |
|  | Multi sector: Road infrastructure |
| 14. Health promotion | Health Promotion |
| 15. Good Hygiene Practices | Good hygiene practices |

Supplement 2. Kenya's "Hub and Spoke" Primary Care Network Model


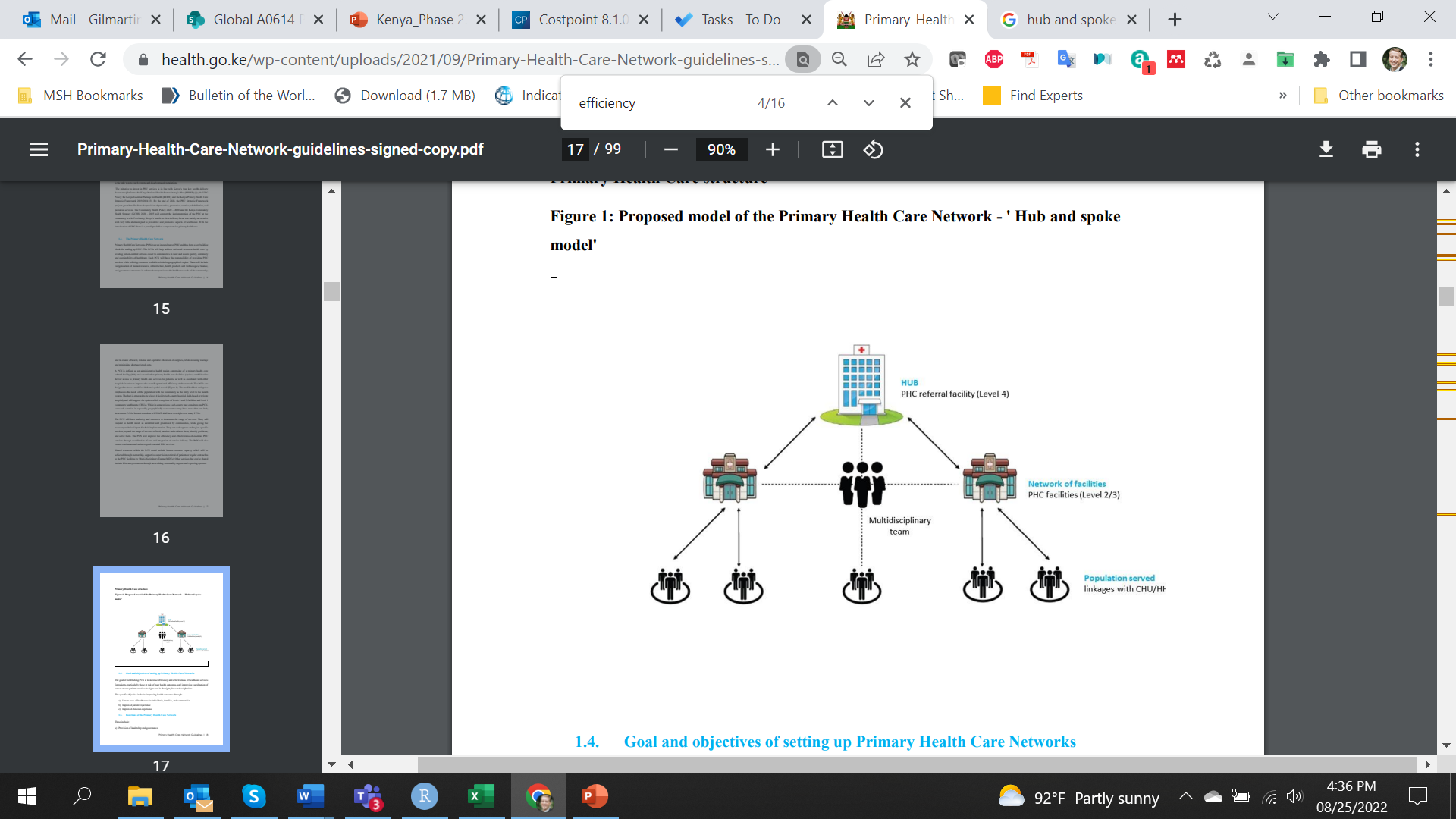


Supplement 3. Map of sampled sub-counties
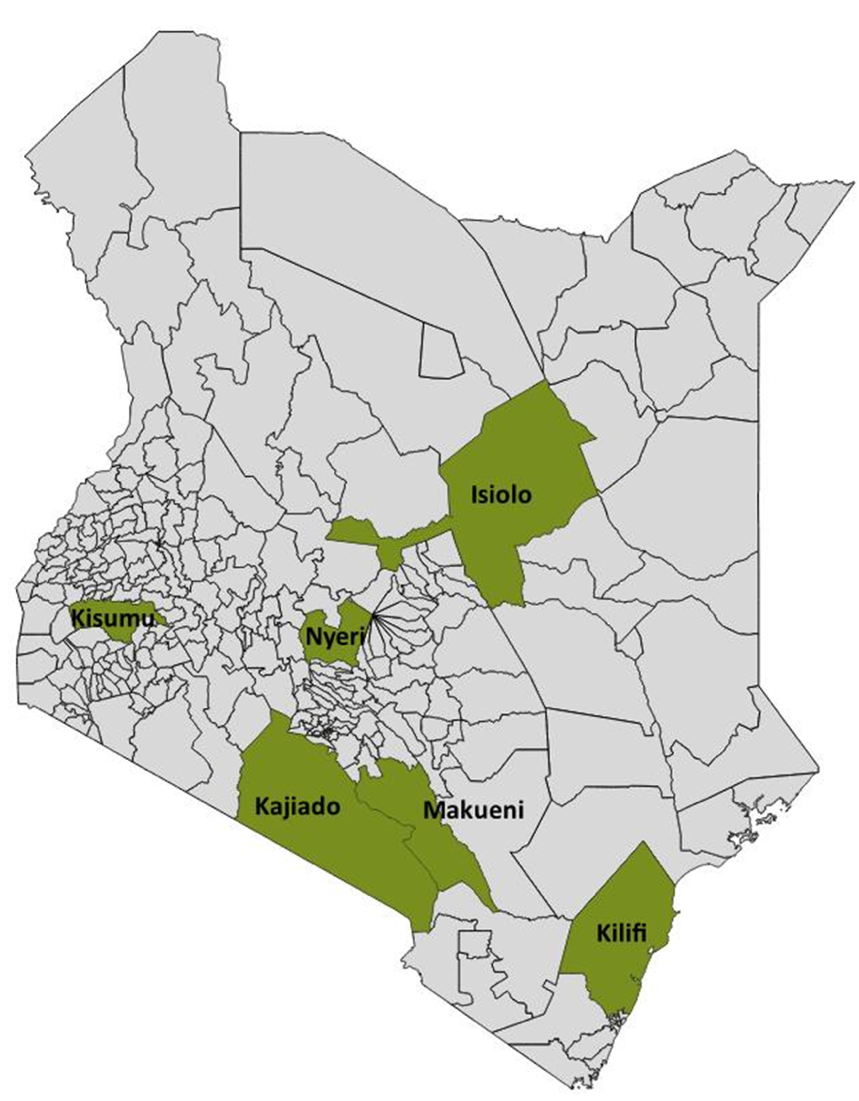


Supplement 4. Actual and normative costs per capita and required increased investment, for different scenarios of clinical time

| **Clinical time (i.e. time facing patients)** | **Actual cost per capita (USD)** | **Normative costs per capita (USD)** | **Required increased investment** |
| --- | --- | --- | --- |
| 50% | 26.6 | 48.3 | 1.8 times |
| 60% | 26.6 | 44.5 | 1.7 times |
| 70% | 26.6 | 41.7 | 1.6 times |
| 80% | 26.6 | 39.7 | 1.5 times |
